# Three distinct hematological malignancies from a single germ cell tumor: a case report 

**DOI:** 10.1186/s13256-020-02558-8

**Published:** 2020-11-17

**Authors:** M. Spencer Chapman, P. C. May, E. Olavarria, E. Nadal Melsio

**Affiliations:** 1 Cancer, Ageing & Somatic Mutation Programme, Wellcome Sanger Institute, Wellcome Genome Campus, Hinxton, Cambridge, UK; 2 Haematology Department, Hammersmith Hospital, Imperial NHS Trust, London, UK; 3 Centre for Haematology, Department of Immunology & Inflammation, Imperial College London, London, UK; 4 SIHMDS, Hammersmith Hospital, Imperial NHS Trust, London, UK

**Keywords:** Germ cell tumor, Teratoma, Mediastinal non seminomatous germ cell tumor, Acute myeloid leukemia, AML, Burkitt’s lymphoma, Klinefelter’s syndrome, Malignant transformation, Clonal evolution

## Abstract

**Background:**

The association between non seminomatous germ cell tumors (GCTs) and hematological malignancies of rare lineage has been described in the literature. In some of these cases there is evidence that the leukemia derives from a pluripotent primitive clone present in the original germ cell tumor.

**Case presentation:**

We present a highly unusual case of a 23-year-old man of South Asian origin with a history of Klinefelter’s syndrome who initially developed mediastinal non seminomatous GCT. Following treatment with surgery and standard chemotherapy he went on to develop three different hematological malignancies of distinct lineages in sequential fashion over a short time period. Despite treatment with multiple intensive chemotherapy regimens and a matched unrelated donor allogeneic stem cell transplant, he died 41 months after initial diagnosis of his GCT and 10 months after the first diagnosis of hematological malignancy.

**Conclusions:**

This is an extreme case that highlights the pluripotency and aggressiveness of these GCT-derived hematological malignancies, and the need for novel therapeutic approaches.

## Background

The majority of male germ cell tumors (GCTs) are located within the testis, however extra-gonadal GCTs represent 1 to 5% of all GCTs and have varied morphology and anatomic location including the anterior mediastinum and retroperitoneum [1]. As a malignancy of primitive cells, GCTs display pluripotentiality for extra-embryonal and embryonal (somatic) differentiation [2, 3]. Among all GCTs, teratomas show the most vivid and varied patterns of somatic differentiation with multiple mature and immature elements [4]. Rarely, teratomas undergo further “malignant transformation” whereby aggressive proliferation of a differentiated non-germ cell tissue develops [5]. This includes neurogenic, epithelial, mesenchymal and hematopoietic malignancies which are often indistinguishable from their spontaneous counterparts, their GCT origin shown only by genetic markers [6, 7]. Where hematopoietic malignancies develop, they are frequently unusual histologic subtypes (e.g. megakaryoblastic leukemia, histiocytic malignancies) and follow an aggressive clinical course [8]. However, they generally remain of one histologic subtype over time. We report an unusual case of a 23-year-old man with Klinefelter’s syndrome and previously treated mediastinal teratoma who went on to develop three different hematological malignancies of distinct lineages in sequential fashion over a short time period.

## Case report

**Fig. 1:**
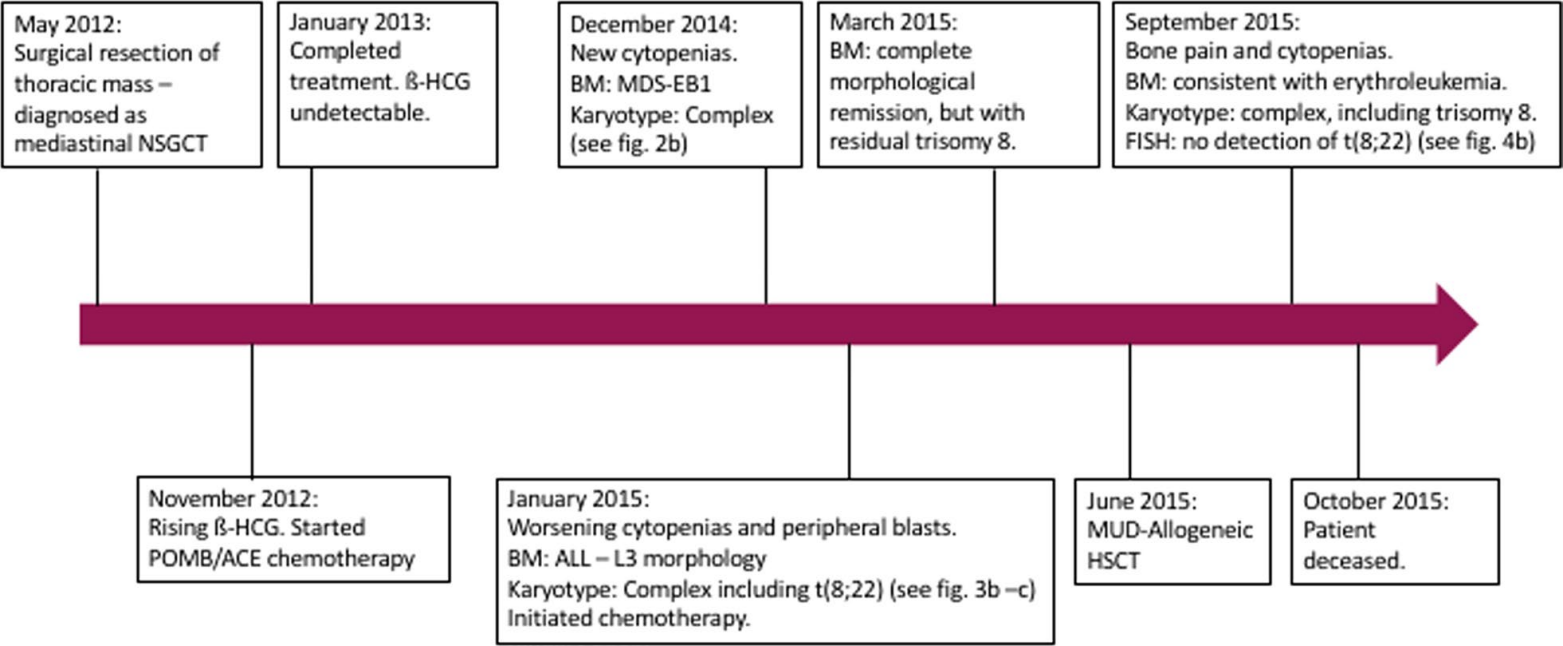
Timeline of patient’s clinical course. *NSGCT* non seminomatous germ cell tumor, *ß-HCG *Beta human chorionic gonadotrophin, *POMB/ACE *chemotherapy regimen (cisplatin, vincristine, methotrexate, bleomycin, daptomycin, cyclophosphamide and etoposide), *BM* bone marrow. *MDS-EB1* myelodysplastic syndrome with excess of blasts-1. *ALL* acute lymphoblastic leukemia, *MUD* matched unrelated donor. *HSCT* hematopoietic stem cell transplantation. *FISH* fluorescence in-situ hybridization

A 23-year-old male of South Asian origin with mosaic Klinefelter syndrome was diagnosed with mediastinal non seminomatous GCT (see Fig. 1 for timeline). Initial management was with surgical resection. At this time the full blood count was normal. Histology confirmed complete excision with narrow margins. Four months later he relapsed with rising levels of beta-human chorionic gonadotrophin (ß-HCG). He was treated with alternating POMB (vincristine 2 mg IV and methotrexate 300 mg/m^2^ over 24 h on day 1, bleomycin 15 mg IV over 24 h on days 2 and 3, cisplatin 120 mg/m^2^ IV over 12 h on day 4) and ACE (dactinomycin 0.5 mg IV on days 1–3, etoposide 100 mg/m^2^ IV on days 1–3, cyclophosphamide 500 mg/m^2^ IV on day 3) chemotherapy, but due to a severe reaction to cisplatin and a presumed treatment-related cerebrovascular accident, cisplatin was substituted with carboplatin. He completed 2 cycles, resulting in biochemical remission.

**Fig. 2:**
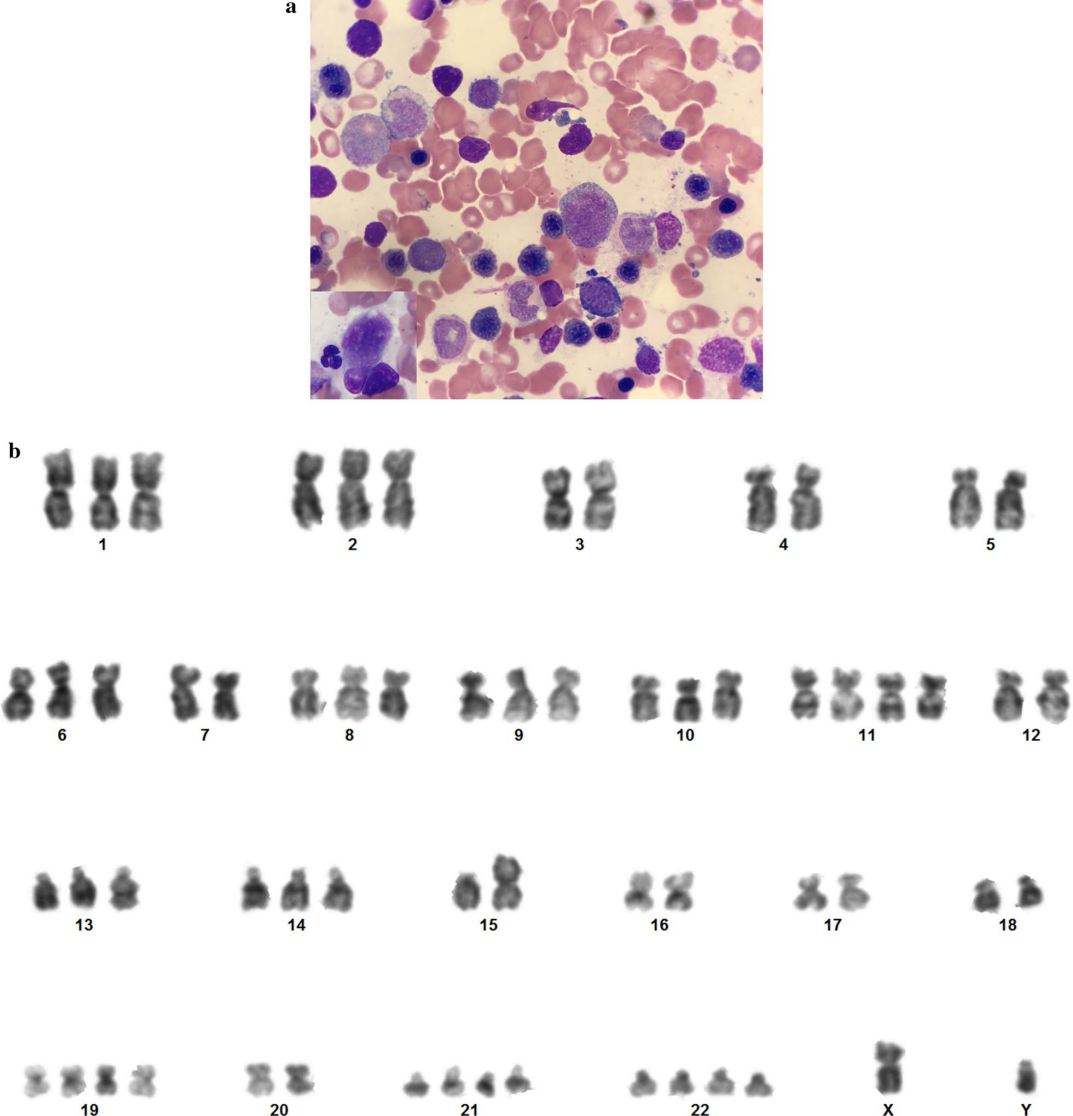
MDS-EB1. **a** BM aspirate showed trilineage dysplasia with excess of blasts (6%) (×*40*). Left lower side inset showing micromegakaryocyte. (×*100*) (Microscope Olympus BX50, camera iPhone SE). **b** G-banded karyogram showing abnormal hypotriploid karyotype, with various numerical gains including trisomy 8 and tetrasomy 22, and a single structural abnormality, an isochromosome comprising two copies of the long arm of chromosome 15

Twenty-three months later he developed thrombocytopenia, macrocytic anaemia, and a raised serum lactate dehydrogenase associated with epistaxis, fatigue and backache. Biochemical markers for GCT remained low. Bone marrow (BM) aspirate showed severe dysplastic changes in all three hematopoietic lineages with 6% myeloblasts classifying him as myelodysplastic syndrome with excess blasts-1 (MDS-EB1) (Fig. 2a). Karyotyping of the BM revealed an abnormal hypotriploid karyotype, with various numerical gains including trisomy 8 and tetrasomy 22, and a single structural abnormality, an isochromosome comprising two copies of the long arm of chromosome 15 (Fig. 2b).

This was an unusual karyotype for MDS and given the high risk of leukemic transformation, he was planned for allogeneic hematopoietic stem cell transplantation (HSCT).

**Fig. 3:**
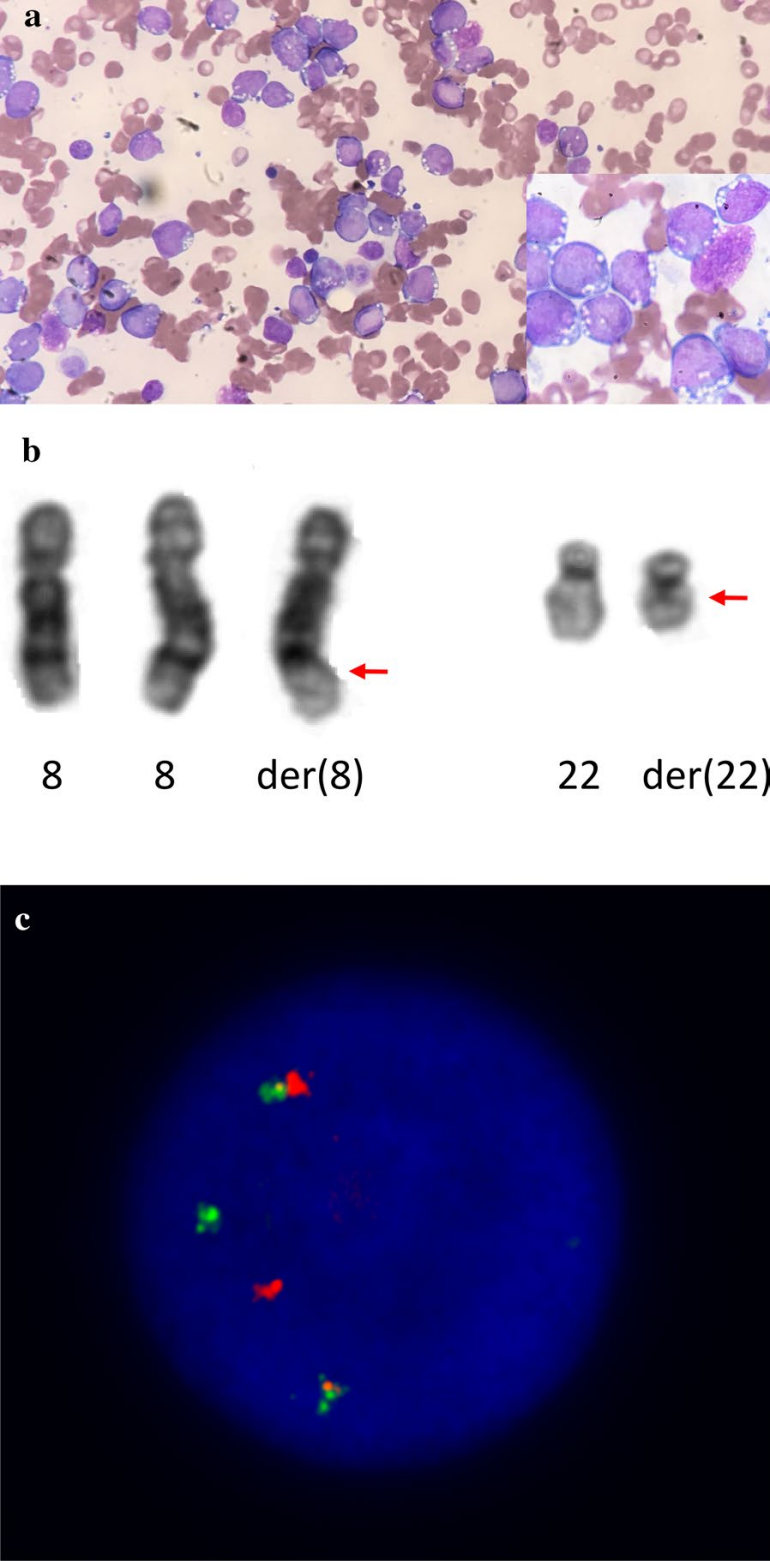
B-ALL. **a** Bone marrow infiltration by blasts with L3 morphology ×20 (*inset *×*100*) (Microscope Olympus BX50, camera iPhone SE). **b** Partial G-banded karyogram showing the *t*(8;22)(q24;q11) with gain of one additional chromosome 8. **c** Fluorescence in situ hybridization using a dual-colour break-apart probe for *MYC* confirmed rearrangement of *MYC* [1R1G2F]

Only 2 weeks after the first BM study and during work-up for HSCT, he developed rapidly worsening cytopenias with peripheral blasts. Repeat BM biopsy now showed infiltration with TdT+ CD10+ abnormal B-cell precursors consistent with acute lymphoblastic leukemia (ALL) with L3 morphology (Fig. 3a). The cytogenetic study revealed an abnormal male karyotype with a new *t*(8; 22) (q24;q11) with *MYC* rearrangement confirmed with FISH, plus other chromosomal abnormalities; a single euploid cell carrying an isochromosome 15 was noted (Fig. 3b, c).

He received induction chemotherapy according to the UK-ALL14 trial protocol [9] (complex regimen including cytarabine, daunorubicin, vincristine, dexamethasone, PEG-asparaginase, methotrexate, cyclophosphamide and mercaptopurine) achieving complete morphologic and cytogenetic remission of ALL but with persistent trisomy 8 in 12% of cells examined by FISH. He proceeded to a matched unrelated donor allogeneic HSCT with reduced intensity conditioning with alemtuzumab (10 mg IV daily on days − 7 to − 3), fludarabine (30 mg/m^2^ IV daily on days − 7 to − 3) and melphalan (140 mg/m^2^ IV on day − 2) and prophylaxis of graft-versus-host disease with post-transplant methotrexate (8 mg/m^2^ IV daily on days + 2, + 4 and + 8). Hematological recovery occurred by day + 40. A BM study on day + 71 post-transplant, performed due to new progressive thrombocytopenia, demonstrated complete cytogenetic remission of the ALL clone, and FISH analysis again showed persistence of trisomy 8 in 9% of examined cells.

**Fig. 4:**
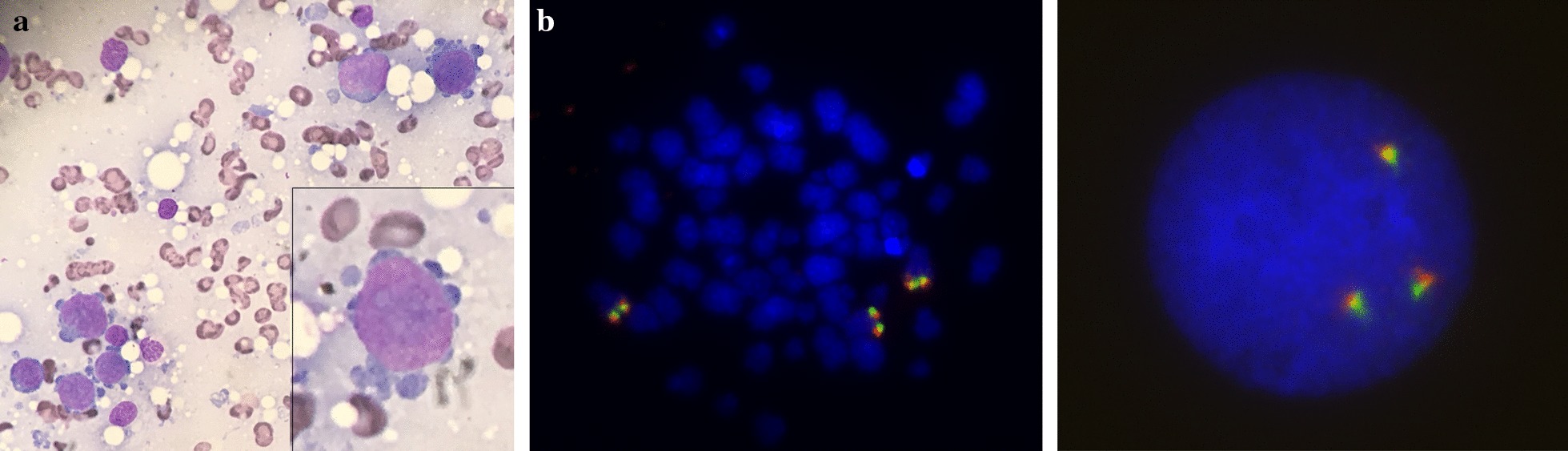
Erythroleukemia. **a** BM trephine roll showing infiltration by abnormal erythroblasts. ×40 (*inset *×*100*) (Microscope Olympus BX50, camera iPhone SE). **b** Metaphase and interphase FISH using the same dual-colour break-apart probe for *MYC* showing no detectable rearrangement of *MYC* but instead an additional signal consistent with trisomy 8 [3F]

One week later he developed tonsillitis, worsening thrombocytopenia necessitating frequent platelet transfusions, and severe opiate-refractory bone pains most marked in the right knee. MRI of the knee showed extensive lytic lesions. Severe sepsis and visual impairment resulting from bilateral retinal hemorrhages then developed. BM study now showed 87% infiltration with abnormal erythroblasts (Fig. 4a) and no evidence of abnormal lymphoid precursors. A diagnosis of pure erythroid leukemia was made. Karyotyping of the BM revealed an abnormal hyperdiploid karyotype, with both structural and numerical abnormalities. FISH for the *MYC* rearrangement remained negative, confirming that this was a new lineage malignancy unrelated to the ALL (Fig. 4b).

FLAG-IDA chemotherapy (fludarabine 30 mg/m^2^ IV for 4 days, cytarabine 2 g/m^2^ IV for 4 days, G-CSF 480 µg from day − 1 until day 5, and idarubicin 10 mg/m^2^ for 3 days) was administered followed by CD34+ stem cell top-up. The patient declined further inpatient treatment and was discharged on day 11 post-chemotherapy. Unfortunately, he died at home 2 weeks later, 41 months after initial diagnosis of GCT, and 10 months after diagnosis of his first hematologic malignancy.

## Discussion

Klinefelter syndrome is the most common constitutional abnormality of the sex chromosomes and is present in an estimated 1/500 to 1/1000 males [10]. Several studies have demonstrated an association between Klinefelter and non seminomatous GCTs as well as breast cancer, lung cancer, and non-Hodgkin lymphoma [11–13]. This increased prevalence may reflect either the role of an extra X chromosome in these tumors or a developmental defect in the migration of germ cells in Klinefelter syndrome [14, 15].

The pluripotency of malignant transformation in GCT has long been recognised both within the original tumor and at metastatic sites [16]. Sarcoma is the most frequent subtype (63%), though adenocarcinoma, primitive neuroectodermal tumor and hematologic neoplasia are all well recognised. The cytogenetic abnormality isochromosome 12p (“i(12p)”) in GCT is well established, with a high prevalence in male GCT. In many cases the clonal origin of metastatic malignancies from the primary GCT is demonstrated by the persistence of this abnormality in the subsequent malignancy [17, 18]. The rare association of hematologic neoplasia and extragonadal germ cell tumors appears to be exclusive to mediastinal non seminomatous GCT with an incidence of 2% in this patient group in one case series of 287 patients [8]. The spectrum of hematologic disease in this setting is unusual, with a predominance of clonal megakaryocytic disorders (AML M7 [French–American–British classification] and MDS with abnormal megakaryocytes) but also mast cell and undifferentiated leukemias (Table 1 [7, 8, 17, 19–30]). Both lymphoblastic and acute erythroid leukemia have been described after or concurrent to GCT but to our knowledge this is the first case where three different sequential hematological malignancies of different lineages have been demonstrated (MDS, ALL and erythroleukemia). It is plausible that the relatively long survival of our patient compared to most of the published cases allowed the development of this wider range of malignancies.

**Table 1:** Features of published cases of hematological malignancies post-germ cell tumor

Patient age (49)	Median		23 years
	Range		11–35 years
Patient sex (63)	Exclusively male		
Anatomic location of prior GCT (75)	Exclusively mediastinal		
Histology of prior GCT (24)	Variable and often mixed, including immature teratoma, yolk-sac tumour, undifferentiated histologies		
Frequency of specific karyotypic abnormalities in hematological malignancy (40)	Presence of i(12p)		68%
	Presence of + 8		29%
	Complex karyotype (≥ 3 structural abnormalities)		48%
Type of hematological malignancy (74)	AML		57%
	Histiocytic disorder		10%
	MDS (with megakaryocytic dysplasia)		11%
	Mast cell leukemia		3%
	ALL		4%
	Acute undifferentiated leukemia		5%
	MPN		7%
	Other		4%
Morphological subtype of AML (where reported) (33)	M2 (Myeloblastic with maturation)		9%
	M3 (Promyelocytic)		3%
	M4 (Myelomonocytic)		24%
	M5 (Monocytic)		9%
	M6 (Erythroblastic)		6%
	M7 (Megakaryoblastic)		48%
Time from GCT diagnosis to hematological malignancy (51)	Median		4 months
	Range		0–47 months
Survival following GCT diagnosis (35)	Median		5 weeks
	Maximum		44 weeks

Recent case reports with allogeneic transplantation and/or novel targeted therapies have had better survival than previous case reports (> 40 weeks, n = 2), however, still none > 1 year

*GCT* germ cell tumor, *i(12p)* isochromosome 12p,* AML* acute myeloid leukemia,* MDS* myelodysplastic syndrome,* ALL* acute lymphoblastic leukemia,* MPN* myeloproliferative neoplasm; M2, M3, M4, M5, M6 and M7 refer to the corresponding French-American-British morphologic classification of acute myeloid leukemia

Number in brackets indicates the number of cases with data on each feature. References for case reports included in table [7, 8, 17, 19–30].

Chemotherapeutic agents used in the initial regimen for this patient can cause therapy-related AML/MDS, with alkylating agents such as cyclophosphamide known to particularly cause monosomy of chromosomes 5 and 7. However previous case series have suggested that the association of hematologic neoplasia following non seminomatous GCT is not therapy-related; the evolution of our case also makes it unlikely [21].

Although cytogenetic analysis of the original tumor was not available in this case to provide evidence that the MDS clone had evolved from the GCT clone, such evolution has been demonstrated in numerous previous cases [8, 17, 21, 24, 26, 31, 32] and seems indeed likely in this instance. On each occasion, cytogenetics revealed numerical and structural abnormalities which showed significant cell-to-cell variation. Our patient developed MDS 30 months after the initial GCT diagnosis. This time-course is somewhat longer than typical with a previous case series suggesting a median time from diagnosis of GCT to hematologic disorder of 5 months (range, 0–32 months) with approximately a third presenting simultaneously [21]. Following this, in a very short time-span, a *MYC* translocation was acquired, driving one leukemic sub-clone towards an aggressive Burkitt-like ALL phenotype. This clone proved responsive to ALL-directed chemotherapy and allogeneic HSCT. However, a third malignant clone with completely different lineage rapidly evolved into an erythroblastic leukemia phenotype. Relapsed disease of a different histologic sub-type despite allogeneic-HSCT is consistent with a previous report of bone marrow transplantation performed in this setting [24]. The dismal prognosis in these rare cases warrants novel therapeutic approaches targeting the multiple clones. In this way Leonard* et al.* recently described a case of GCT with concomitant AML which achieved a partial sustained remission for 6 months with targeted therapy after* in vitro* assays demonstrated an *NRAS* mutation common to both the GCT and the concomitant erythroleukemia [26].

## Conclusions

GCTs have shown the pluripotent capacity of transforming into malignancies of diverse origin; most commonly sarcoma. However mediastinal GCT can transform into unusual and diverse hematologic malignancies, likely from an initial “hematologic malignancy precursor” derived from the GCT [32]. Our case demonstrates the rapid sequential transformation into three distinct hematological malignancies: MDS, B-ALL with *MYC* rearrangement and pure erythroid leukemia despite aggressive treatment. As in previously reported cases, allogeneic-HSCT likely prolonged survival but was ultimately ineffective at preventing malignant transformation. Hematological diseases that arise from non seminomatous GCT show an aggressive and fatal course despite intensive treatment; novel therapeutic approaches targeting the pluripotent clone are needed to improve the outcome of these patients.

## Data Availability

The key data supporting the conclusions of this article are included within the article. Further data including full cytogenetic reports are available on request.

## Abbreviations

ACE: Chemotherapy regimen for germ cell tumors, consisting of dactinomycin, etoposide and cyclophosphamide (see main text)
ALL: Acute lymphoblastic leukemia
AML: Acute myeloid leukemia
B-ALL: B-cell acute lymphoblastic leukemia
BM: Bone marrow
ß-HCG: Beta-human chorionic gonadotrophin
FISH: Fluorescence in-situ hybridization
FLAG-IDA: Chemotherapy regimen for acute leukemias consisting of fludarabine, cytarabine, G-CSF and idarubicin (see main text)
G-CSF: Granulocyte colony stimulating factor
GCT: Germ cell tumor
HSCT: Hematopoietic stem cell transplantation
i(12p): Isochromosome 12p
IV: Intra-venous
MDS: Myelodysplastic syndrome
MDS, EB-1: Myelodysplastic syndrome with an excess of blasts (5–10%)
MPN: Myeloproliferative neoplasm
MRI: Magnetic resonance imaging
MUD: Matched unrelated donor
NSGCT: Non seminomatous germ cell tumor
POMB: Chemotherapy regimen for germ cell tumors, consisting of cisplatin, vincristine, methotrexate and bleomycin (see main text)
